# Cognitive function is associated with the progression of non-tremor motor symptoms in Parkinson’s disease

**DOI:** 10.3389/fnagi.2026.1765860

**Published:** 2026-02-12

**Authors:** Melissa C. Gibbs, Aminette D’Souza, Charalampos Sotirakis, Trevor J. Crawford, Chrystalina A. Antoniades

**Affiliations:** 1 NeuroMetrology Lab, Nuffield Department of Clinical Neurosciences, John Radcliffe Hospital, University of Oxford, Oxford, United Kingdom; 2 Department of Psychology, Lancaster University, Lancaster, United Kingdom

**Keywords:** cognition, longitudinal, MoCA, motor, non-tremor, OxQUIP, Parkinson’s disease

## Abstract

**Background:**

Parkinson’s disease (PD) is characterized by motor impairment which consists of tremor and non-tremor symptoms. Cognitive function may overlap with specific aspects of voluntary movement and action initiation. This study aims to investigate associations between global cognition and the severity and longitudinal progression of tremor and non-tremor motor symptoms in PD.

**Methods:**

As part of the Oxford Quantification in Parkinsonism (OxQUIP) study, 84 participants with PD were tested over seven visits at three-month intervals. At each visit, participants completed standardized global cognitive (MoCA) and motor (MDS-UPDRS-III) assessments. Tremor and non-tremor motor subscores were derived from corresponding MDS-UPDRS-III items. Linear mixed-effects models were calculated to analyze the effect of global cognition at baseline on the progression of (i) overall motor impairment, (ii) non-tremor motor symptoms, and (iii) tremor symptoms.

**Results:**

We did not find an association between MoCA scores and MDS-UPDRS-III severity, but there was a significant interaction between global cognition and the progression of motor impairment (*p* = 0.005). Lower MoCA scores were linked with steeper progression of non-tremor motor symptoms (*p* < 0.001), but not tremor symptoms (*p* = 0.380).

**Conclusion:**

Global cognition at baseline is associated with the progression, but not severity, of motor impairment in PD; this finding is specific to non-tremor and not tremor motor symptoms. While both motor subdomains are known to be linked with dysfunction of sub-cortical circuits, non-tremor symptoms may also be influenced by disrupted cognitive inputs. Our results highlight the potential value of incorporating cognitive tools to complement motor examination in PD assessment.

## 1 Introduction

Parkinson’s disease (PD) is a progressive neurodegenerative disorder involving the loss of dopaminergic neurons and alpha-synuclein aggregation primarily in the substantia nigra. The clinical presentation is characterized by several cardinal motor symptoms, including tremor, bradykinesia, rigidity, and disturbed gait and posture (Postuma et al., 2015). This broad range of motor symptoms may be more effectively evaluated when classified into distinct tremor and non-tremor subdomains, which are known to differ in terms of presentation, progression, and associations with non-motor symptoms of PD (Jankovic et al., 1990; Tosin et al., 2020).

Cognitive dysfunction is a common non-motor feature in PD, with reports suggesting a prevalence of mild cognitive impairment in approximately 25.8% of people with PD (Aarsland et al., 2021). There is clinical heterogeneity in the presentation of cognitive deficits in PD, as impairments may be observed across multiple cognitive domains (Murakami and Hanajima, 2023; Kehagia et al., 2010). In clinical practice, cognitive function is often evaluated using brief, easily-administered, and well-validated global cognitive tools – such as the Montreal Cognitive Assessment (MoCA) – which provide an overall index of cognitive performance (Skorvanek et al., 2018). Dopaminergic pathology is linked with the presentation of these cognitive symptoms, with greater loss of dopaminergic neurons associated with global cognitive impairment (Aarsland et al., 2021). Given shared neurobiological mechanisms underpinning cognitive and motor decline, the interaction between symptoms may help explain the heterogeneity observed in PD.

Non-tremor motor behaviors, such as gait and hand movements, share functional overlap with cognitive functions involved in initiating and controlling voluntary movement. Previous work has demonstrated associations between global cognition and a range of non-tremor motor symptoms, including bradykinesia, rigidity, and gait and postural abnormalities (Murakami et al., 2013). For example, the front-visuospatial network is involved in the flexible adaptation of motor behavior and has been linked to the regulation of both gait velocity and variability in neuroimaging studies (Amboni et al., 2013). Structural and functional changes in cognitive brain regions have been associated with worse non-tremor motor symptoms in PD (Schneider et al., 2015).

In contrast, tremor symptoms are considered to be less influenced by cognitive function (Domellof et al., 2011; Schneider et al., 2015). Cognitive impairment in PD is less commonly associated with tremor-dominant phenotypes than with non-tremor motor symptoms, such as postural instability and gait disturbances (Poletti et al., 2012). Neuroanatomical evidence shows that fronto-striatal pathways are relatively spared in tremor-dominant patients (Barbagallo et al., 2017). Instead, tremor has been linked to cerebellar-driven dysfunction within cerebello-thalamo-circuits (Zhong et al., 2022). These differences may underlie the selective associations observed between cognitive function and non-tremor motor symptoms in PD. However, most studies in this area have been cross-sectional; the cognitive contributions to motor subdomains are yet to be fully examined longitudinally.

This study aims to investigate whether cognitive function is associated with the severity and longitudinal progression of motor symptoms in PD. We focus on global cognition – a comprehensive measure of overall cognitive status – as this is often assessed in clinical practice and previous work links multiple cognitive domains to motor outcomes in PD. We aimed to examine the effect of global cognition on (i) overall motor impairment, (ii) non-tremor motor symptoms, and (iii) tremor motor symptoms. We hypothesize that global cognition is associated with the severity and progression of overall motor impairment in PD and that this association would be specific to non-tremor, rather than tremor, motor symptoms.

## 2 Materials and methods

### 2.1 Participants

**Figure 1:**
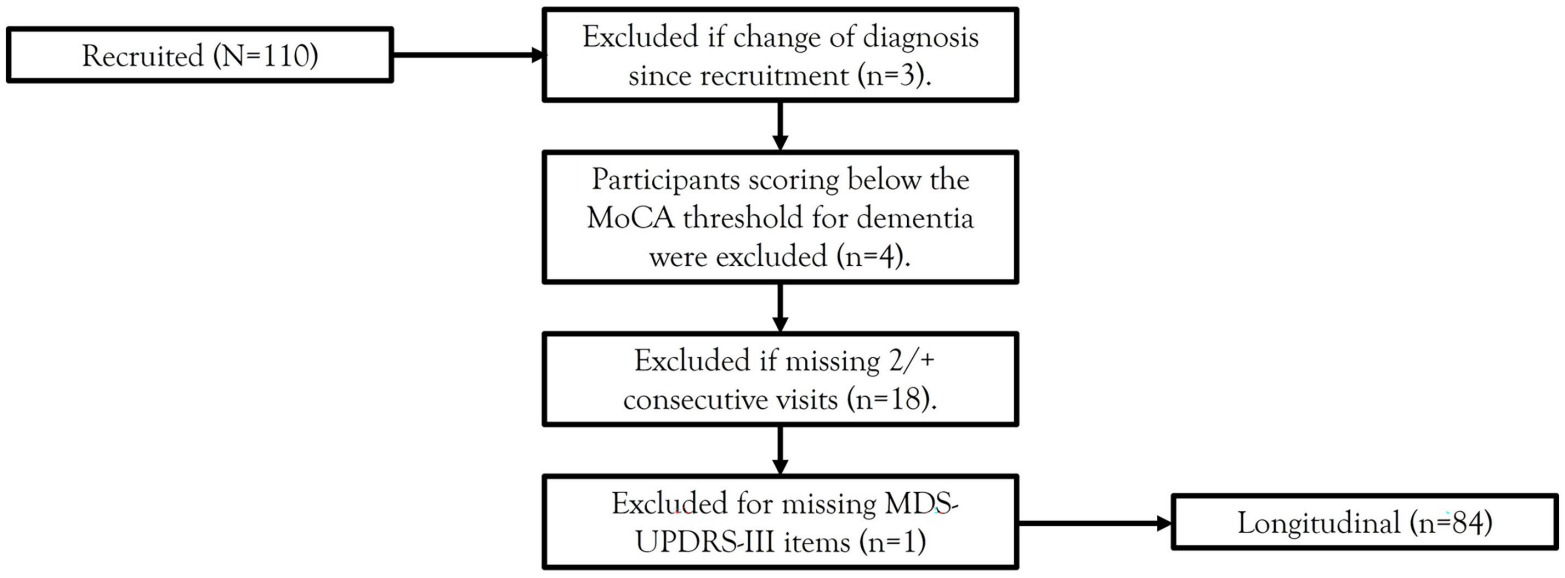
Flowchart of sample selection criteria. MoCA, Montreal Cognitive Assessment; MDS-UPDRS-III, Movement Disorders Society-Unified Parkinson’s Disease Rating Scale-Part III.

Participants were recruited via the Oxford Quantification in Parkinsonism (OxQUIP) study conducted at the John Radcliffe Hospital in Oxford, UK. The OxQUIP study is a longitudinal observational study of neurophysiological and neuropsychological biomarkers in Parkinsonian disorders, approved by the Research Ethics Committee (16/SW/0262). All participants provided informed written consent prior to participation. A total of 110 participants with a diagnosis of idiopathic PD according to the 1988 UK PD Brain Bank Criteria and Movement Disorders Society (MDS) criteria (Postuma et al., 2015) were recruited. Participants were excluded from the present analysis if they had a change of diagnosis over the course of the study, scored below the MoCA threshold for dementia (<21) (Dalrymple-Alford et al., 2010), missed 2 or more consecutive visits, or consecutively missed specific items over longitudinal visits (see Figure 1 for a flowchart of the sample selection). The final sample included 84 PD participants.

### 2.2 Experimental procedure

Participants attended in-person assessments every 3 months for a total of seven visits. During each visit, a battery of clinical tests was administered, including assessments of motor performance [Movement Disorders Society-Unified Parkinson’s Disease Rating Scale-Part III (MDS-UPDRS-III); Postuma et al., 2015] and global cognition (MoCA; Nasreddine et al., 2005). MoCA is a standardized clinical assessment of global cognition which is well-suited to screening for cognitive impairment in PD (Dalrymple-Alford et al., 2010). All patients were tested during an ON-state.

### 2.3 Data preparation

Data were analyzed using RStudio (Version 4.3.3). To prepare the longitudinal MDS-UPDRS-III dataset, linear interpolation was used to impute missing values where there were one or two non-consecutive missed visits before the seventh visit. An average value was calculated using the visit data prior to and following the missing data point. Where data was missing for the seventh visit, items from visit six were carried forward. If a patient attended a visit but individual items were incomplete, an average was calculated using item scores prior to and following the missing item.

MDS-UPDRS-III items were divided into subscores comprising tremor (3.15–3.18) and non-tremor (3.1–3.14) items based on a previous factor analysis of 6,298 PD participants (Tosin et al., 2020). These subscores were analyzed as proportions to account for differences in the total number of items between both subdomains (e.g., Tremor subscore proportion = Sum of Tremor scores / Maximum possible Tremor score).

In the present work, we analyze MoCA scores which were measured at baseline, as it is influenced by practice effects over repeated assessments (Cooley et al., 2015). For descriptive statistics and visualization, baseline MoCA scores were used to categorize patients as either cognitively normal (CN; MoCA ≥ 26) or cognitively impaired (CI; MoCA = 21–25) based on previously validated thresholds of cognitive impairment in PD (Dalrymple-Alford et al., 2010). In statistical analyses, MoCA scores were analyzed as a continuous measure to capture variation.

### 2.4 Statistical analysis

The normality of distributions was checked using the Shapiro–Wilk test. As motor data was non-normally distributed on most visits, we used linear mixed-effects models which are robust to normality violations and allow us to examine the linear relationship between global cognition and motor function across visits. Separate models were calculated to analyze effects on the severity and progression of (i) total MDS-UPDRS-III scores, (ii) MDS-UPDRS-III non-tremor subscores, and (iii) MDS-UPDRS-III tremor subscores. The fixed effects of Visit, MoCA scores, and their interaction term were entered into all models; a participant-level random effect was also included to account for inter-individual variability. A power calculation determined that we had a 98% probability of detecting a significant effect at *α* = 0.05, assuming an effect size of R^2^ = −0.446 based on previous findings (Murakami et al., 2013). For models where significant effects were found, further sensitivity analyses were conducted to determine whether observed effects were robust to the inclusion of covariates, i.e., age, sex, time since diagnosis in years, educational attainment, levodopa equivalent daily dose (LEDD) (see Supplementary materials). Post-hoc inspections confirmed that all model assumptions were met, including homoscedasticity and normality of residuals. Cook’s distance tests did not reveal significant influences of outliers on model estimates.

## 3 Results

### 3.1 Sample demographics

Demographics and baseline scores are summarized in Table 1. No significant differences were observed between cognitive status groups (PD-CN: MoCA score ≥ 26, PD-CI: MoCA score = 21–25) across demographic and motor variables at baseline.

**Table 1:** Sample demographics at baseline for cognitively normal (PD-CN) and cognitively impaired (PD-CI) groups.

Sample characteristics	PD-CN (*n* = 67) mean (SD)	PD-CI (*n* = 17) mean (SD)	*p*-value
Age, years	64.40(7.36)	67.53(8.35)	0.200
Sex, Male: Female	36:31	11:6	0.589
Time since diagnosis, years	4.25(4.18)	5.02(4.32)	0.358
LEDD	459.40(393.57)	466.54(387.82)	0.964
Education, Above HE: Below HE	22:44	5:12	0.924
Total MDS-UPDRS-III	23.34(11.54)	26.47(15.95)	0.536
Tremor subscore	5.16(3.83)	5.06(4.66)	0.884
Non-tremor subscore	18.18(10.04)	21.42(13.61)	0.490
H&Y, range	1.61(1–3)	1.65(1–4)	0.138
MMSE	28.75(1.21)	27.88(1.58)	0.036*
MoCA	27.90(1.39)	23.82(1.29)	<0.001***

LEDD, Levodopa Equivalent Daily Dose; HE, Higher Education (BSc and above); MDS-UPDRS-III, Movement Disorders Society-Unified Parkinson’s Disease Rating Scale-Part III; H&Y, Hoehn and Yahr; MMSE, Mini Mental State Examination; MoCA, Montreal Cognitive Assessment. MDS-UPDRS-III scores were taken as proportions to account for missing values. *p-*values calculated using Chi-square test for gender, education, and H&Y; Wilcox test used for other demographic and clinical variables. Where full Opicapone dosage not present, LEDD estimated by multiplying total LEDD by 1.33 (*n* = 1). *p < 0.05, ***p < 0.001.

### 3.2 Baseline cognition is associated with overall motor symptom progression

**Figure 2:**
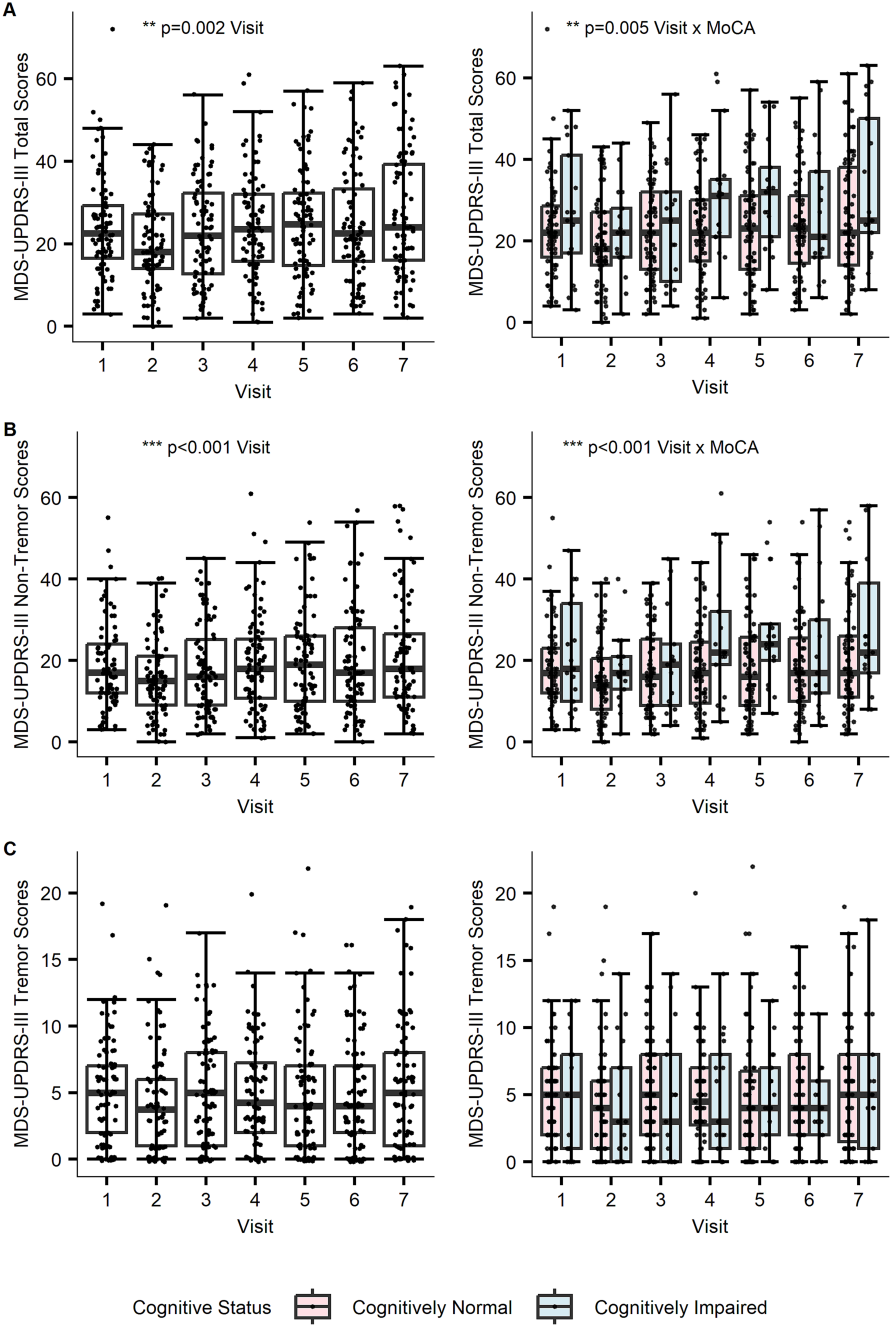
Effects of global cognition on longitudinal motor scores for separate **(A)** total MDS-UPDRS-III, **(B)** non-tremor, and **(C)** tremor models. **(A)** The plot on the left shows that overall motor scores progressed significantly across visits (*p* = 0.002), with MoCA influencing the trajectory of progression as seen on the right (*p* = 0.005). **(B)** Non-tremor subscores progressed significantly across visits (*p* < 0.001), with MoCA influencing the trajectory of progression (*p* < 0.001). **(C)** Tremor subscores did not progress across visits (*p* = 0.454), with MoCA having no impact on progression (*p* = 0.380). Cognitive status groups determined from MoCA scores at baseline (cognitively normal: MoCA ≥ 26, cognitively impaired: MoCA = 21–25). Potential outliers did not influence model estimates.

First, we aimed to determine whether global cognition influenced longitudinal changes in overall motor impairment. Motor symptoms (measured using the MDS-UPDRS-III rating scale) showed significant progression across visits [*F*(1,502) = 10.33, *p* = 0.002]. Global cognitive function did not independently influence the severity of motor scores [*F*(1,142) = 0.24, *p* = 0.623]. However, there was a significant interaction between baseline MoCA scores and visit, with lower global cognitive function linked with a more rapid progression in motor impairment [*F*(1,502) = 8.34, *p* = 0.005] (Figure 2). A sensitivity analysis showed that the interaction between baseline MoCA scores and visit [*F*(1, 502) = 8.34, *p* = 0.004] was robust to the inclusion of covariates (See Supplementary Table 1).

### 3.3 Baseline cognition is associated with non-tremor motor symptom progression

Next, we divided motor impairment into subdomains to determine whether global cognition specifically influenced non-tremor motor symptoms. Replicating the pattern above, non-tremor motor symptoms progressed significantly across visits [*F*(1,502) = 15.68, *p* < 0.001]. Global cognitive function did not have a main effect on the severity of non-tremor subscores [*F*(1,134) = 0.15, *p* = 0.701], but it interacted with the progression of non-tremor motor symptoms [*F*(1,502) = 13.16, *p* < 0.001]. Lower global cognitive function was linked with greater progression of non-tremor motor symptoms (Figure 2). A sensitivity analysis showed that the interaction between baseline MoCA scores and visit [*F*(1, 502) = 13.16, *p* < 0.001] was robust to the inclusion of covariates (See Supplementary Table 2).

### 3.4 Baseline cognition is not associated with tremor motor symptoms

Finally, we aimed to determine whether global cognition influenced tremor motor symptoms. Tremor symptoms did not progress across longitudinal visits [*F*(1,502) = 0.56, *p* = 0.454]. Global cognitive function did not influence MDS-UPDRS-III tremor severity [*F*(1,125) = 0.17, *p* = 0.677] or interact with progression [*F*(1,502) = 0.77, *p* = 0.380] (Figure 2).

## 4 Discussion

This study investigated the relationship between global cognition and the severity and longitudinal progression of motor impairment in a cohort of PD participants. We found that cognition was associated with the progression, but not severity, of overall motor symptoms: lower MoCA scores were linked with greater progression in MDS-UPDRS-III motor performance. The separate examination of the non-tremor and tremor symptoms revealed that the link between global cognition and motor symptoms was specific to the non-tremor subdomain. Lower MoCA scores were associated with greater longitudinal decline of the non-tremor motor symptoms, while MoCA did not influence tremor symptoms.

Previous literature has highlighted significant longitudinal associations between global cognitive impairment and global motor dysfunction in PD (Rodriguez-Antiguedad et al., 2025). Cognitive impairment in PD may reflect broader network-level dysfunction which also manifests across motor functions. While we found that cognition influences the longitudinal trajectory of motor impairment, we did not find an effect on the severity of impairment. This finding contrasts with a previous cross-sectional study which demonstrated a correlation between MoCA scores and overall motor function on the UPDRS scale (Murakami et al., 2013). However, the previous work recruited both PD with mild cognitive impairment (PD-MCI) and PD with dementia (PDD) and did not separate these groups in their analysis. In our sample, we excluded participants scoring below the MoCA threshold for dementia, to avoid conflating these diagnostic categories. The mechanisms underlying cognitive impairment in PD-MCI and PDD are known to differ: compared with PD without cognitive impairment and PD-MCI, PDD is characterized by more widespread dopaminergic pathology across frontal, parietal, and temporal cortical regions, as well as *β*-amyloid and tau deposition which may be indicative of coexisting Alzheimer’s disease (AD) pathology in many patients (Aarsland et al., 2021). Given these neuropathophysiological differences between PD diagnostic categories, it becomes important to study these groups independently in larger cohorts, as it is likely that the strength and direction of cognition-motor associations vary between groups (Rodriguez-Antiguedad et al., 2025). Another explanation for the discrepancy in findings may be sample characteristics, as the previous study recruited an older cohort with a longer disease duration (Murakami et al., 2013); both factors are known to influence symptom severity in PD (Nasreddine et al., 2005; Aarsland et al., 2021). Furthermore, longitudinal approaches applied in our study provide deeper insight into how relationships between symptoms change over time compared to a single cross-sectional snapshot of the disease.

A key finding of our study is a selective association between global cognition and non-tremor motor progression in PD. This can be interpreted in the context of cortico-basal ganglia (CBG) pathways involved in the cognitive control of voluntary movement. The motor loop is a relatively simple pathway, which modulates reflexive and unconscious motor behaviors via feedback circuits through the basal ganglia to target areas in motor cortices (Alexander et al., 1986). Inputs from prefrontal cognitive brain areas are required to exert control over more complex facets of actions selection. This communication occurs via a series of parallel, functionally segregated circuits called CBG loops, which have been well documented to subserve different aspects of motor control alongside behavior (Alexander et al., 1986). In PD, we observe a characteristic breakdown in the transfer of information from prefrontal to motor loops (Antoniades et al., 2015). Consistent with CBG models, frontal executive dysfunction has been linked with non-tremor motor impairment (Kelly et al., 2015; Schneider et al., 2015). Cognitive impairment may exacerbate reduced interloop information transfer, thereby accelerating the decline of non-tremor motor symptoms over the course of the disease.

In line with our results, previous neuropsychological work has not detected significant associations between global cognitive function and tremor severity (Domellof et al., 2011; Murakami et al., 2013; Schneider et al., 2015). Tremor dominance is likely more associated with cerebellar-driven dysfunction of cerebello-thalamo-circuits (Zhong et al., 2022) compared to impaired voluntary motor behaviors arising from dysfunctional cognitive control over CBG circuitry (Alexander et al., 1986). Indeed, fronto-striatal pathways are relatively spared in patients experiencing tremor-dominant symptoms (Barbagallo et al., 2017). Subtyping studies have also detected neuroanatomical differences, with significant reductions in the volume of grey matter in cognitive brain areas in postural instability and gait dominant patients compared to tremor-dominant patients (Rosenberg-Katz et al., 2013). Consistent with previous work (Xia and Mao, 2012), we found that tremor symptoms did not progress over time. In our cohort, all PD participants were taking dopaminergic medication by the end of the study. It is possible that medication may differentially contribute to the progression of specific motor symptoms due to differences in underlying pathophysiology.

Beyond PD, associations between cognition and motor function are also observed in other neurodegenerative diseases and healthy aging. Gait impairments (e.g., slower gait speed and increased gait variability) are linked with poorer performance on global and domain-specific cognitive tasks in aging, AD, and Progressive Supranuclear Palsy (PSP) (Amboni et al., 2013; Morris et al., 2016; D'Souza et al., 2025). Although Parkinsonian disorders are typically characterized by motor impairment and AD by prominent cognitive symptoms, these conditions often present with overlapping deficits, likely reflecting degeneration within shared brain networks supporting motor and cognitive function. However, it is important to note that evidence from AD and healthy aging largely focuses on the gait domain (Morris et al., 2016) rather than broader motor functions as examined in our paper, making it difficult to generalize our findings more widely.

The nature of cognitive impairment in PD is another important point to consider. Cognitive profiles in PD and the mechanisms that underlie them are known to be heterogeneous (Murakami and Hanajima, 2023; Kehagia et al., 2010). Studies which focus on domain-specific cognitive impairments in PD, such as executive dysfunction, report selective associations with motor domains (Schneider et al., 2015; Moustafa et al., 2016). For example, patients with predominantly akinetic-rigid symptoms display greater attentional and executive deficits compared to tremor-dominant patients, despite no observed differences in global cognition between groups (Moretti et al., 2020). This may also explain why, in our study, we find that global cognition interacts with motor progression but does not influence the level of motor symptom severity. Whilst MoCA is an easily administered and widely used clinical screening tool for cognitive status, it may lack the sensitivity and specificity to detect cognitive deficits which are more isolated in nature and to disentangle deficits arising from coexisting pathologies. Furthermore, MoCA is susceptible to practice effects over repeated assessments (Cooley et al., 2015), and MoCA subdomains are associated with floor and ceiling effects (Koshimoto et al., 2023). To validate and extend our findings, future work should utilize neuropsychological tests which are domain-specific and sensitive to early decline and longitudinal changes in PD, such as the Symbol Digital Modalities Test (Cao et al., 2024; Oltra et al., 2022) and the Frontal Assessment Battery (Bezdicek et al., 2017). This would provide deeper insight into how distinct cognitive profiles in PD may be associated with motor function and progression. Recent work has shown that the development of digital biomarkers, both for the assessment of cognitive and motor function, may hold potential for improving diagnosis and longitudinal monitoring of symptoms in PD (De Vos et al., 2020; Sotirakis et al., 2023, 2024; Craig et al., 2025).

The results discussed here should be considered in light of certain limitations. Previous research has demonstrated that MoCA scores are influenced by age and educational attainment (Nasreddine et al., 2005). While our sensitivity analyses revealed that the relationship between cognition and motor progression was robust to the inclusion of these covariates, future work should recruit larger, heterogeneous cohorts to explore the independent effects of these demographic factors. It is possible that PD medication (i.e., levodopa) may also influence the relationship between motor symptoms and cognition (Amboni et al., 2018). All patients in our cohort were tested during an ON-state and there were no differences in medication dosage between cognitively impaired and unimpaired groups in our sample. Further studies comparing medication states are needed to improve our understanding of the mechanistic pathways linking motor and cognitive symptoms in PD. To extend our findings, future research should compare neurodegenerative disease models to disentangle the complex relationship between cognition and motor function. This could lead to improvements in differential diagnostic tools for conditions that present similarly in early disease stages despite pathophysiological differences, such as PD and PSP.

## 5 Conclusion

Our study demonstrates a complex interplay between cognitive and motor impairment in PD. Global cognitive function at baseline is associated with the progression, but not severity, of motor impairment; this finding is specific to non-tremor motor symptoms in PD and not tremor symptoms. These results highlight the potential of cognitive prognostic biomarkers for motor progression in PD: a standardized and widely used clinical tool, such as MoCA, may serve as a useful baseline measure to evaluate the progression of specific motor symptoms. Further work is needed to develop more sensitive and specific cognitive tools to monitor the longitudinal progression of symptoms in PD. Our results highlight the potential value of incorporating cognitive tools to complement motor examination in PD assessment.

## Data Availability

The raw data supporting the conclusions of this article will be made available by the authors, without undue reservation.
