# Supplementary material for: Cognitive function is associated with the progression of non-tremor motor symptoms in Parkinson’s disease
Source: Front Aging Neurosci. 2026 Feb 12;18:1765860. doi: 10.3389/fnagi.2026.1765860 (PMC12935966; doi:10.3389/fnagi.2026.1765860)
Supplement: Supplementary file 1 [file Data_Sheet_1.pdf]

Supplementary Materials

Supplementary Table 1. Effects of Visit, Cognition, and Covariates on MDS-UPDRS-III Total motor scores.

	β	SE	t-stat	p-value
Visit	0.051	0.016	3.214	0.001**
MoCA	0.001	0.005	0.159	0.874
Age	0.002	0.001	1.320	0.190
Sex	- 0.035	0.017	-2.051	0.044*
Time since diagnosis, years	0.006	0.002	2.338	0.022*
Educational Attainment	0.001	0.011	0.127	0.900
LEDD	-0.001	0.001	-0.133	0.894
Visit x MoCA	-0.002	0.001	-2.887	0.004**

Note. Mixed-Effects Model: MDS-UPDRS-III Total Motor Score ~ Visit * MoCA + Age + Sex + Time since diagnosis in years + Educational Attainment + LEDD + 1|Subject. MDS-UPDRS-III = Movement Disorders Society-Unified Parkinson's Disease Rating Scale-Part III, MoCA = Montreal Cognitive Assessment, LEDD = Levodopa Equivalent Daily Dose. * = p<0.05, ** = p<0.005.

Supplementary Table 2. Effects of Visit, Cognition, and Covariates on MDS-UPDRS-III Non-tremor motor scores.

	β	SE	t-stat	p-value
Visit	0.079	0.020	3.960	<.001***
MoCA	0.002	0.006	0.359	0.720
Age	0.003	-0.002	-1.669	0.099
Sex	-0.046	0.023	-2.044	0.044*
Time since diagnosis, years	0.006	0.003	1.685	0.096
Educational Attainment	-0.005	0.014	-0.330	0.743
LEDD	0.001	0.001	0.809	0.421
Visit x MoCA	-0.003	0.001	-3.628	<.001***

Note. Mixed-Effects Model: MDS-UPDRS-III Non-Tremor Motor Score ~ Visit * MoCA + Age + Sex + Time since diagnosis in years + Educational Attainment + LEDD + 1|Subject. MDS-UPDRS-III = Movement Disorders Society-Unified Parkinson's Disease Rating Scale-Part III, MoCA = Montreal Cognitive Assessment, LEDD = Levodopa Equivalent Daily Dose. * = p<0.05, *** = p<0.001.
